# Assessing DNA Degradation through Differential Amplification Efficiency of Total Human and Human Male DNA in a Forensic qPCR Assay

**DOI:** 10.3390/genes15050622

**Published:** 2024-05-14

**Authors:** Elena Chierto, Serena Aneli, Nicola Nocco, Alessia Riem, Martina Onofri, Eugenia Carnevali, Carlo Robino

**Affiliations:** 1 Department of Public Health Sciences and Pediatrics, University of Turin, 10126 Turin, Italy; 2 Forensic Sciences Laboratory, Section of Legal Medicine, Department of Medicine and Surgery, Santa Maria Hospital, University of Perugia, 05100 Terni, Italyeugeniacarnevali@gmail.com (E.C.); 3 Section of Legal Medicine, Department of Medicine and Surgery, University of Perugia, 06123 Perugia, Italy

**Keywords:** forensic genetics, DNA degradation, qPCR, FFPE samples, paternity testing

## Abstract

The assessment of degradation is crucial for the analysis of human DNA samples isolated from forensic specimens. Forensic quantitative PCR (qPCR) assays can include multiple targets of varying amplicon size that display differential amplification efficiency, and thus different concentrations, in the presence of degradation. The possibility of deriving information on DNA degradation was evaluated in a forensic qPCR assay not specifically designed to detect DNA fragmentation, the Plexor HY (Promega), by calculating the ratio between the estimated concentrations of autosomal (99 bp) and Y-chromosomal (133 bp) targets (“[Auto]/[Y]”). The [Auto]/[Y] ratio measured in 57 formalin-fixed, paraffin-embedded samples was compared to a quality score (QS) calculated for corresponding STR profiles using quantitative data (allele peak height). A statistically significant inverse correlation was observed between [Auto]/[Y] and QS (R = −0.65, *p* < 0.001). The [Auto]/[Y] values were highly correlated (R = 0.75, *p* < 0.001) with the “[Auto]/[D]” values obtained using the PowerQuant (Promega) assay, expressly designed to detect DNA degradation through simultaneous quantification of a short (Auto) and a long (D) autosomal target. These results indicate that it is possible to estimate DNA degradation in male samples through Plexor HY data and suggest an alternative strategy for laboratories lacking the equipment required for the assessment of DNA integrity through dedicated qPCR assays.

## 1. Introduction

The quantity and integrity of human DNA in forensic samples are often impossible to predict, as they are influenced by multiple factors that are largely not known a priori, such as the time since the deposition of a stain and the subsequent effect of environmental agents, both physical (solar irradiation, humidity) and biological (microbial activity), which can progressively fragment nucleic acids, thus reducing the final DNA yield [1].

For this reason, the precise determination of the concentration of human DNA isolated from forensic samples has become a crucial step in the analytical workflow. In standard human identification procedures consisting of multiplex PCR amplification of short tandem repeat (STR) polymorphisms followed by capillary electrophoresis (CE), the quantification step firstly enables traces without human DNA to be discarded, consequently saving laboratory time and reagents. Secondly, it allows the identification of samples with low numbers of DNA copies, in which characteristic stochastic artefacts, of the drop-out or drop-in type, may occur during PCR [2]. Moreover, quantification permits the accurate dosing of the amount of template DNA to be included in the amplification reaction to avoid possible electrophoretic artefacts caused by DNA overload, such as non-template addition causing split peaks and the potential genotyping errors of STR microvariants [3].

Traditional DNA quantification methods that measure UV absorption or fluorescence emitted by DNA-intercalating dyes are not sufficiently sensitive for forensic traces, and equally importantly, they are not specific to human DNA [4]. This led to the development of molecular methods specifically devised for human identification purposes, initially based on the probe hybridization of human DNA either blotted on a membrane [5] or in liquid phase [6].

However, such solutions were quickly rendered obsolete by the emergence of the Real-Time PCR (RT-PCR) technique, or quantitative PCR (qPCR) [7]. qPCR takes advantage of fluorescently labelled primers/probes designed to amplify/hybridize a DNA target of interest. Depending on the type of qPCR assay, the accumulation of the PCR product is accompanied by a progressive increase/decrease in fluorescence emission, which can be monitored by the RT-PCR instrument with extreme sensitivity and within a wide dynamic range. In forensic quantitation assays, the measurement of fluorescence emission during the PCR exponential phase using human DNA samples with known concentrations allows a standard curve to be derived that can be used for the quantification of unknown samples amplified in the same experiment [8]. The increased sensitivity of forensic qPCR assays can be achieved by adopting multicopy elements dispersed in the human genome as amplification targets [8].

In recent years, the technical evolution of RT-PCR instruments has enabled the multiple detection of an ever-growing number of fluorophores. This gave way to the possibility of enriching forensic qPCR assays with additional dye-labelled probes/primers, thus increasing the amount of useful information that can be obtained from analyzed DNA. The simultaneous qPCR amplification of autosomal and Y-chromosomal targets allows the ratio between total and male DNA to be calculated [9,10]. In mixed male/female samples, such as those typically found in sexual assault cases, such data are crucial for the choice between standard STR and Y-STR genotyping [11]. The inclusion of qPCR primers/probes targeting an internal PCR control (IPC) enables the detection of the co-extraction of PCR inhibitors with DNA [12] and to identify cases where further DNA purification is needed to prevent the failure of STR amplification [13]. A recent advancement is the development of RT-PCR systems that can assess the degradation status of a tested DNA sample [14,15,16]. This is achieved through the simultaneous qPCR amplification of two human DNA targets, located on autosomes, with different sizes in bp. In a degraded DNA sample, the larger target is more prone to DNA degradation, leading to a depletion of the template available for amplification compared to the smaller target. As a consequence, the ratio of human DNA concentration measured by qPCR for the smaller and larger targets will reflect the level of degradation in the sample. This now enables forensic investigators to identify, before STR profiling [17], low-template DNA (LT-DNA) samples at high risk of PCR stochastic artefacts, and to consequently adopt appropriate analytical strategies, which may include increased PCR cycling [2]; enhanced CE settings [18]; splitting of the DNA sample over PCR replicates [19]; and a combination of multiple STR typing kits in order to increase the number of genotyped loci [20].

A possible limitation of the last generation of commercial qPCR assays that combine total human detection and human male detection with inhibition and degradation information, such as the Quantifiler™ Trio (ThermoFisher Scientific, Waltham, MA, USA) [14], PowerQuant^®^ (Promega, Madison, WI, USA) [15], Investigator^®^ Quantiplex^®^ Pro (Qiagen, Hilden, Germany) [21] and InnoQuant^®^ HY (InnoGenomics Technologies, LLC, New Orleans, LA, USA) [22] kits, is that they are designed and optimized for use with only a limited number of RT-PCR instruments. In this study, we investigated the ability of a commercial qPCR assay for the quantification of human DNA (Plexor HY, Promega), compatible with a wide range of RT-PCR instruments, to provide useful information on DNA integrity even in the absence of specific additional primers/probes for the assessment of degradation. The Plexor HY includes a 99 bp target located on human chromosome 17 for the quantification of total human DNA and a 133 bp target on the Y chromosome for the quantification of male DNA [10]. It is possible to hypothesize that, in the presence of fragmented DNA, the amplification of the small autosomal target will be favoured compared to the long Y-chromosome target and that, therefore, the ratio between the concentration of total human and human male DNA can provide an estimate of the DNA degradation status in the sample. To test this hypothesis, we evaluated the qPCR results and the quality of STR profiles obtained in a collection of formalin-fixed, paraffin-embedded (FFPE) samples of male subjects analyzed by the laboratory in the context of deficiency paternity investigations. The ability of formalin to fragment and modify DNA [23], thus reducing the amount of the template available for PCR amplification, makes FFPE samples an ideal substrate for the study of DNA degradation by qPCR.

## 2. Materials and Methods

### 2.1. FFPE Samples

FFPE samples (*n* = 57) pertaining to seventeen different deficiency paternity cases assigned to the laboratory in the period 2013–2023 were considered in the study. Samples consisted of both neoplastic and non-neoplastic tissues processed in twelve different histopathology services. The years of fixation ranged between 1996 and 2022. For two FFPE samples from a single case, the year of fixation and histopathology service were unknown, and information regarding possible neoplastic nature was missing. The general characteristics of the FFPE samples are summarized in Table S1. FFPE samples of interest are indicated hereafter as the combination of case and sample numbers reported in Table S1.

### 2.2. DNA Extraction

Two to three sections of tissue, 5–10 µm thick, were cut via a microtome from each FFPE sample and deparaffinized using Deparaffinization Solution (Qiagen) followed by DNA purification with a QIAamp DNA FFPE Tissue kit (Qiagen) performed either manually (2013–2021) or automatically (2022–2023) with a QIACube Connect (Qiagen).

### 2.3. DNA Quantitation

DNA extracts were originally quantified at the time of paternity testing with the Plexor HY system (Promega, Madison, WI, USA) using a CFX96 Touch (Bio-Rad Laboratories, Hercules, CA, USA) RT-PCR instrument. To create the quantitation standard curve, seven serial 1:5 dilutions of the provided DNA standard in the range of 50 ng/μL to 3.2 pg/μL were prepared and amplified in duplicate. qPCR results were analyzed using Plexor Analysis software (Promega) to estimate the concentrations of total human (autosomal) and human male (Y-chromosomal) targets from which the [Auto]/[Y] ratio was derived. The possible presence of PCR inhibitors was assessed by means of IPC. In particular, inhibition was assumed in a DNA sample when the IPC cycle threshold (C_T_) showed a shift ≥2 cycles compared to the C_T_ value of a DNA standard with an equivalent concentration of total human DNA [10]. After anonymization (only non-identifying information summarized in Table S1 was retained), the same DNA extracts, which had meanwhile been stored and frozen at −20 °C, were newly quantified in a single experiment conducted in 2024 using the PowerQuant system (Promega) on a 7500 Real-Time PCR System (ThermoFisher Scientific). The quantitation standard curve included four serial 1:25 dilutions of the provided DNA standard in the range of 50 ng/μL to 3.2 pg/μL amplified in duplicate. The PowerQuant Analysis software (version 1.0.0.0) (Promega) was then used to calculate the concentrations of total human (autosomal) and human male (Y-chromosomal) targets, and the [Auto]/[D] ratio, which compares the concentration of the autosomal target (84 bp) with that of a degradation control target, mapping at the same locus as the autosomal target, characterized by longer amplicon size (294 bp). In PowerQuant, the inhibition of a DNA sample was defined as a ≥0.3 shift between the quantification cycle (Cq) of IPC and the Cq of a DNA standard with an equivalent concentration of total human DNA [15].

### 2.4. STR Amplification, Genotyping, and Determination of Quality Score of STR Profiles

The STR amplification kits used for paternity testing in the selected FFPE samples varied and included AmpF*l*STR Identifiler Plus (ThermoFisher Scientific) (*n* = 7), Investigator ESSplex (Qiagen) (*n* = 4), Investigator ESSplex SE QS (Qiagen) (*n* = 5), PowerPlex ESI 17 (Promega) (*n* = 16) and PowerPlex ESX 17 (Promega) (*n* = 23) (Table S1). In all cases, a DNA input of 0.5 ng was PCR-amplified, applying standard conditions according to the manufacturer’s instructions. For samples with suboptimal DNA concentration, maximum DNA input volume according to each STR kit amplification protocol was added to the reaction mix. STR genotyping was performed by CE using the ABI Prism 310 Genetic Analyzer (Applied Biosystems, Waltham, MA, USA) (2013–2018) and the SeqStudio Genetic Analyzer (ThermoFisher Scientific) (2019–2023). Data analysis was carried out with GeneMapper software (version 5) (ThermoFisher Scientific, Waltham, MA, USA). The limit of detection (LDT), separating reported alleles from electrophoretic baseline noise, was set at 50 relative fluorescence units (RFU) for all STR kits and CE instruments.

All the STR amplification kits used for the genotyping of FFPE samples were preliminarily subjected to experiments conducted according to Gill et al. [24] in order to estimate the probability of drop-out through logistic regression. This allowed us to calculate, for each STR kit, a specific RFU value corresponding to the 5% stochastic threshold [25]. The probability that observed alleles with a peak height above this RFU threshold may have an unseen companion allele that dropped out below LDT was less than 5%. In each STR profile, the largest amplicon product with a peak height above the 5% stochastic threshold was identified and its length in bp was used as a numerical quality score (QS) to express the potential loss of information due to DNA degradation. The smaller the QS value, the higher the degradation of the STR profile data, and vice versa. An example of QS determination is provided for the electropherogram depicted in Figure S1. Depending on the QS value, DNA profiles were classified as highly degraded (QS ≤ 250 bp) or mildly degraded (QS > 250 bp). The 250 bp cut-off was chosen considering the current availability of “miniSTR” amplification kits, using primers repositioned as close as possible to the STR region with maximum amplicon range ≤250 bp, specifically designed for the genotyping of degraded DNA [26].

### 2.5. Statistical Analysis

Statistical comparisons and correlations of data distributions were computed using the Spearman correlation coefficient and the Wilcoxon test, respectively, while the paired-samples Wilcoxon test was used to compare paired data. A statistical significance threshold of *p* < 0.05 was applied. The performance of [Auto]/[Y] and [Auto]/[D] values for distinguishing FFPE samples affected by different degrees of degradation were assessed by receiver operating characteristic (ROC) curve analysis and reported as the area under the curve (AUC). Finally, cut-offs maximizing sensitivity and specificity were calculated.

All statistical analyses were conducted using R statistical software (version 4.2.1) [27] and Python 3.7.

## 3. Results

### 3.1. Total Human and Human Male DNA Concentration in FFPE Samples

**Figure 1:**
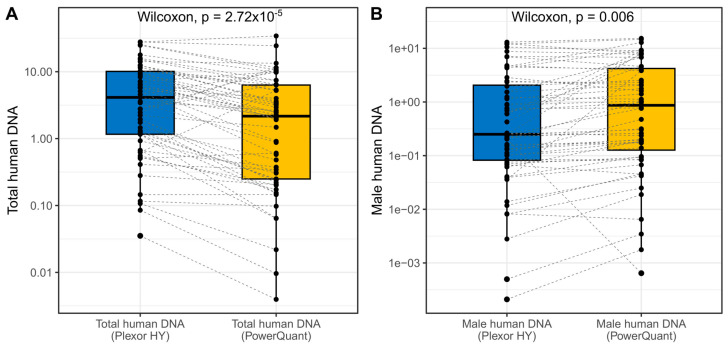
Distribution and comparison of total human DNA (**A**) and human male DNA (**B**) concentrations (ng/µL, log_10_ scale) in FFPE samples as determined with Plexor HY (blue boxplots) and PowerQuant (orange boxplots). Individual FFPE samples are depicted as black dots. Dashed lines connect the results of corresponding FFPE samples analyzed with the two different qPCR assays.

Total human DNA and human male DNA concentrations and IPC results determined in each FFPE sample by Plexor HY and PowerQuant qPCR assays are listed in Table S1. With the exception of three samples (11-1, 13-1, 13-2), PCR efficiency for total human DNA in historical quantification experiments was within the expected optimal range (slope of standard curve between −3.1 and −3.7). An ideal slope (−3.0/−3.6) was always observed for human male DNA. An almost double human male DNA concentration compared to total human DNA concentration was observed, both with Plexor HY and PowerQuant, in FFPE sample 13-2. No Y-specific amplification signal was detected for a single sample with PowerQuant (6-2). For the same sample, male DNA quantification with Plexor HY was close to the lower range of DNA standards used for calibration (0.005 ng/µL). No evidence of inhibition was observed in any FFPE sample based on IPC C_T_ delay ≥ 2 (Plexor HY) or IPC Cq shift ≥ 0.3 (PowerQuant). The distributions of total human DNA and human male DNA concentrations in the overall FFPE sample set obtained with the two qPCR assays are displayed in Figure 1.

As can be seen, Plexor HY showed a significant tendency to overestimate total human DNA concentration in relation to PowerQuant (paired-samples Wilcoxon, *p* < 0.001). The opposite was seen for human male DNA (*p* = 0.006). In both qPCR assays, no significant differences in DNA concentrations were observed when samples were divided into two groups according to neoplastic and non-neoplastic nature. Total human DNA concentration calculated with Plexor HY and year of fixation of FFPE samples were not correlated, while a weak positive correlation (R = 0.30, *p* = 0.0260) was observed between total human DNA and year of fixation for PowerQuant, with older FFPE samples generally showing reduced DNA yield compared to the most recent ones (Figure S2A,B). Even though quantification experiments using PowerQuant were conducted between 1 and 11 years after the initial Plexor HY analyses, with a linear regression model it was possible to observe that, while a reduction in total human DNA concentration was present in more recent quantification results, this was not statistically significant (β = −0.125, *p* = 0.486).

### 3.2. [Auto]/[Y] and [Auto]/[D] Values in FFPE Samples

**Figure 2:**
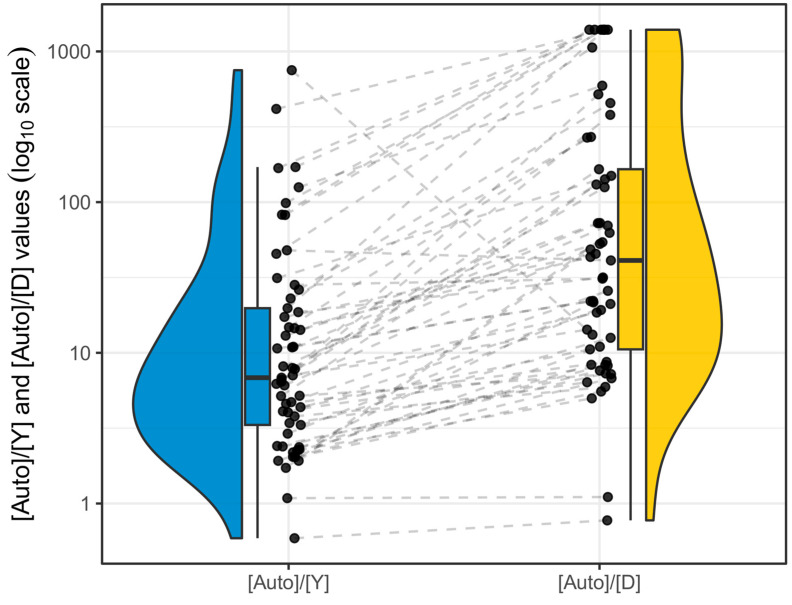
Distribution and comparison of Plexor HY [Auto]/[Y] (left, blue colour) and PowerQuant [Auto]/[D] (right, orange colour) values. Individual FFPE samples are depicted as black dots. Dashed lines connect the corresponding FFPE samples analyzed with the two different qPCR assays.

The [Auto]/[Y] (Plexor HY) and [Auto]/[D] (PowerQuant) values determined in each FFPE sample can be found in Table S1 and their distribution across the whole FFPE sample set is displayed in Figure 2.

Notably, a <1 [Auto]/[D] value was obtained for sample 13-2, which also showed inverted autosomal/Y DNA concentrations in both the qPCR assays. PowerQuant Analysis software assigned “undetermined” [Auto]/[D] values to six FFPE samples (14-1, 14-2, and all four samples of case 17) due to the failed amplification of the degradation control target. Since the aforementioned samples did not display anomalous IPC shifts, qPCR results could only be explained by severe degradation of DNA. Consequently, the maximum [Auto]/[D] value observed overall in other FFPE specimens was assigned to these samples in Figure 2 and in further calculations. Similarly to what was observed for total human DNA and human male DNA concentrations, no significant differences in [Auto]/[Y] and [Auto]/[D] values were found between neoplastic and non-neoplastic FFPE samples. On the other hand, significant inverse correlations were present between [Auto]/[Y] (R = −0.62, *p* < 0.001) and [Auto]/[D] (R = −0.53, *p* < 0.001) values and year of fixation (Figure S2C,D), and between [Auto]/[Y] (R = −0.29, *p* = 0.027) and [Auto]/[D] (R = −0.42, *p*-value = 0.001) values and total human DNA concentration (Figure S3), with older and less concentrated FFPE DNA samples showing increased overamplification of the shorter autosomal target compared to the longer Y-chromosomal (Plexor HY) or degradation control (PowerQuant) targets.

**Figure 3:**
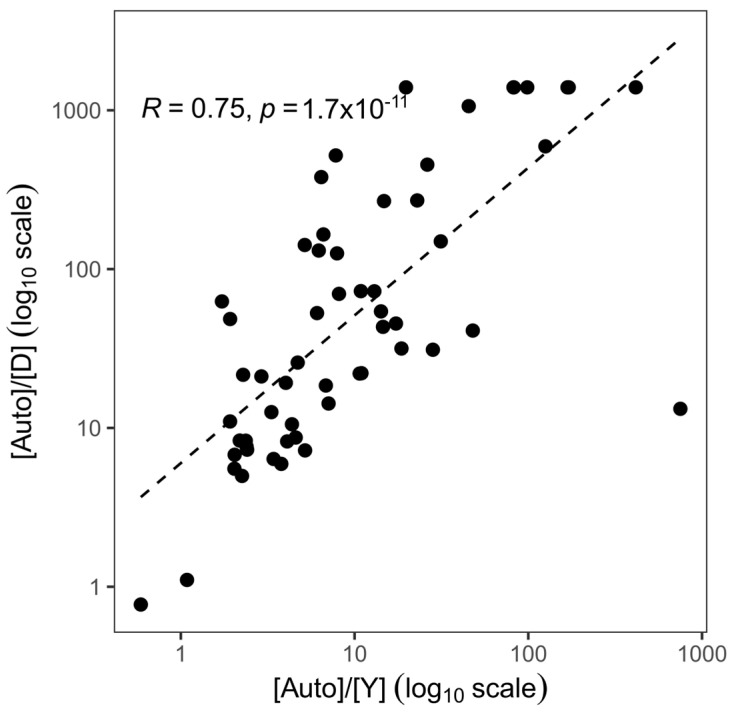
Correlation between [Auto]/[Y] measured by Plexor HY (x axis) and [Auto]/[D] measured by PowerQuant (y axis).

Finally, [Auto]/[Y] and [Auto]/[D] values appeared to be strongly correlated (R = 0.75, *p* < 0.001) (Figure 3).

### 3.3. STR Profile QS Values

**Figure 4:**
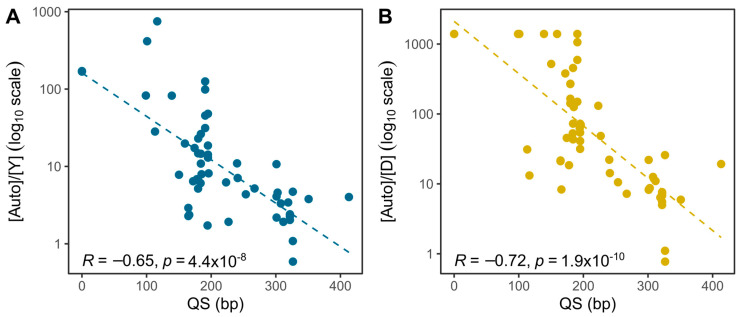
Correlation between QS (bp) of STR profiles (x axis) and (**A**) [Auto]/[Y] measured by Plexor HY; (**B**) [Auto]/[D] measured by PowerQuant.

QS values calculated according to 5% stochastic threshold and logistic regression model in the STR profiles of each FFPE sample are listed in Table S1. No significant differences in QS values were observed between neoplastic and non-neoplastic FFPE samples, while a significant positive correlation was present between the QS value and year of fixation (R = 0.57, *p* < 0.001) (Figure S2E), with more recent FFPE specimens displaying higher QS values. A significant inverse correlation was found between QS and [Auto]/[Y] (R = −0.65, *p* < 0.001) and [Auto]/[D] (R = −0.72, *p* < 0.001) values (Figure 4), with the lower quality of STR profiles in FFPE samples characterized by increased overamplification of the shorter autosomal target compared to the longer Y-chromosomal (Plexor HY) or degradation control (PowerQuant) targets.

### 3.4. Inferring STR Profiling Success in FFPE Samples by [Auto]/[Y] and [Auto]/[D] Values

STR profiling results and related QS values were used to determine [Auto]/[Y] and [Auto]/[D] thresholds that could be applied to predict the success of STR typing based on qPCR data. FFPE samples’ DNA profiles were first classified, according to QS value, as highly degraded (QS ≤ 250 bp) or mildly degraded (QS > 250 bp).

**Figure 5:**
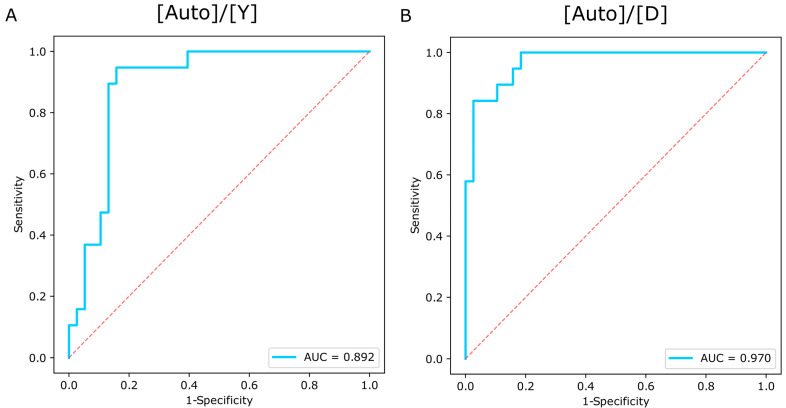
ROC curves and AUC values describing the performance of (**A**) [Auto]/[Y] (Plexor HY) and (**B**) [Auto]/[D] when distinguishing between highly (QS ≤ 250 bp) and mildly (QS > 250 bp) degraded FFPE DNA samples.

Both [Auto]/[Y] and [Auto]/[D] showed good diagnostic accuracy for the discrimination between highly and mildly degraded samples (AUC values = 0.892 and 0.970 for [Auto]/[Y] and [Auto]/[D], respectively; Figure 5).

For [Auto]/[Y], the threshold maximizing classification success between highly and mildly degraded STR profiles was 5.19, with 94.7% sensitivity and 84.2% specificity. For [Auto]/[D], a threshold of 12.58 with 84.2% sensitivity and 97.4% specificity was calculated.

Finally, in deficiency paternity cases in which multiple FFPE specimens were available (*n* = 12), the possibility of using [Auto]/[Y] and [Auto]/[D] values and the calculated threshold maximizing classification success as a tool to identify the DNA sample least subjected to the loss of STR profile information was assessed (Table 1).

In 75% of the cases, the FFPE sample with the lowest [Auto]/[Y] value was also the one with the highest QS. In a single case (12), the choice of an alternative FFPE sample associated with a higher [Auto]/[Y] value led to a conspicuous gain of information, with the longest peak with an RFU height above the 5% stochastic threshold being 413 bp, compared to 227 bp in the sample with the lowest [Auto]/[Y]. Only 11 cases could be considered for PowerQuant, since in a single case the [Auto]/[D] values of all FFPE specimens were undetermined. In 64% of the cases, the sample with the lowest [Auto]/[D] was also the one with the highest QS. Only in case 12, the same identified in Plexor HY, was the loss of information deemed as relevant, with a 159 bp difference between the longest peak with an RFU height above the 5% stochastic threshold in the selected sample (254 bp) and that of the “best” sample (413 bp). Notably, this was also the only case in which, while one or more samples with an [Auto]/[D] value below the threshold predicting degradation (12.58) were present, the STR profile with the highest QS belonged to an FFPE sample with [Auto]/[D] value > 12.58. Only in 27% of comparable cases did [Auto]/[Y] and [Auto]/[D] identify the same FFPE sample as the most promising for STR typing. However, even in discordant cases, gain/loss in terms of QS measured in STR profiles selected with the two methods was limited, never exceeding +/− 32 bp.

## 4. Discussion

DNA quantitation results obtained in FFPE samples with the two qPCR assays showed a relative overestimation of total human DNA and a relative underestimation of human male DNA with Plexor HY compared to PowerQuant. The latter observation could be partly explained by the effect of different target copy numbers in the two assays. Both qPCR methods target multicopy regions, represented in Plexor HY by the RNU2 [28] and TSPY [29] genes arranged in tandem repeats on the long arm of chromosomes 17 and Y, respectively. PowerQuant uses undisclosed high-copy-number proprietary targets: one autosomal (84 bp), and two (81 and 136 bp) located on the Y chromosome [15]. There is evidence that the adoption of two rather than one multicopy Y-chromosomal target, coupled with the reduced amplicon size of the shorter Y-target, can increase the sensitivity of PowerQuant [15,30] and therefore explain the comparatively reduced concentrations of human male DNA estimated by Plexor HY in FFPE samples.

With equal number of autosomal targets, the effect of different assay designs on the quantification of total human DNA remains difficult to assess. As the size of target amplicons remains the most obvious reason for different quantification results detected across forensic qPCR assays in reference DNA standards and artificially degraded samples [31,32,33], the overestimation of the total human DNA target (99 bp) by Plexor HY compared to PowerQuant (84 bp) was unexpected. This observation could be partly explained by the different detection chemistries. While PowerQuant is based on standard hydrolysis probe technology [34], Plexor HY is a primer-based assay in which pairing between a dye-labelled modified base (isocytosine), positioned at the 5′ end of one RT-PCR primer, and a synthetic quencher molecule included in the reaction mix (dabcyl-iso-dGTP) causes a gradual reduction in fluorescence as amplification progresses [35]. Similar counterintuitive results have been previously observed in studies conducted on casework samples comparing Plexor HY and qPCR assays using hydrolysis probes comparable to PowerQuant [36].

Another factor to take into account is that PowerQuant quantification experiments were performed after a 1–11-year interval since the original Plexor HY analysis. In the intervening period, DNA samples obtained from FFPE samples had been subjected to −20 °C long-term storage. However, it is possible that, despite cold storage, some DNA loss may have occurred due to freeze-thawing, retention in polypropylene tubes, evaporation, or denaturation [37]. Accordingly, a slight, albeit not significant, reduction (0.125 ng/µL per year) was observed for total human DNA in the PowerQuant quantification of archival FFPE DNA samples.

The observed differential amplification efficiency between short ([Auto]) and long ([Y] in Plexor HY, [D] in PowerQuant) qPCR targets reflects the DNA fragmentation induced by formalin fixation. Formalin-driven DNA alterations include the cleavage of glycosidic bonds and the generation of apurinic and apyrimidinic sites susceptible to breakage [38]. The process is known to be time-dependent [39] and intensified in an acidic environment, favoured by the use of unbuffered formalin, which promotes the accumulation of formic acid over time [40]. Both [Auto]/[Y] and [Auto]/[D] values were positively correlated with the year of fixation. This result reflects the wide temporal range of fixation years (1996–2022) of FFPE samples included in the study. It is likely that, during this time interval, progressive steps including a reduced fixation period and adoption of buffered formalin were adopted by processing laboratories, leading to a general improvement in DNA quality for downstream molecular applications [41].

Plexor HY [Auto]/[Y] and PowerQuant [Auto]/[D] values appeared to be strongly correlated in the tested FFPE samples, indicating that [Auto]/[Y] can be used as a meaningful alternative to systems specifically devised to detect DNA degradation. Previous electrophoretic studies of DNA isolated from archival FFPE specimens clearly pointed out that median fragment size is generally below 1 kb, and that most DNA fragments are ≤200 bp [42,43]. Accordingly, RT-PCR studies using pairs of targets of varying size (<100 bp, 100–300 bp, >300 bp) have shown that in FFPE samples, qPCR assays combining targets with limited differential length, located slightly above and below the 100 bp threshold similarly to Plexor HY (99–133 bp), and of a size far below that of the degradation targets of current forensic qPCR assays (200–350 bp) [32], can be effectively used to assess DNA fragmentation [44,45]. The use of qPCR assays combining pairs of shorter targets, despite the reduced differential length in bp, may even provide a more realistic assessment of DNA integrity by preventing the possible amplification failure frequently observed when using longer degradation targets [46,47]. This is especially relevant in the forensic context where, through the use of STR amplification kits aptly designed to detect short DNA fragments [44] and the combination of multiple PCR kits with different primer designs [18,45], it is now possible to seize relevant evidentiary information even from highly fragmented DNA samples.

The final aim of this study was therefore to put the DNA quantitation and STR profiling results into relation. Several methods have been proposed to assess the informativity of STR profiles by the inspection of electrophoretic results, the simplest being the percentage of reportable alleles by the number of expected alleles in the reference profile [48]. In this case, the reference profiles of the FFPE sample donors were not available. Moreover, it is now generally accepted that the quantitative information derived from CE (allelic peak height measured in RFU) is a better indicator of STR profile quality, since it enables the likely magnitude of stochastic effects that may complicate the interpretation of results in LT-DNA samples to be inferred [17]. A numeric quality parameter (QS) was calculated from the electrophoretic results of the FFPE samples that were inversely correlated with both [Auto]/[Y] and [Auto]/[D], indicating that Plexor HY data can be used for the assessment of expected STR typing success, with a remarkable AUC value of 0.892. While QS is a continuous variable, a decisional threshold was set at 250 bp, corresponding to the maximum amplicon range of the currently available “miniSTR” kits [26] and indicating highly degraded samples. In such samples, the amplification of the larger degradation target of PowerQuant (294 bp) is expected to be hampered and, therefore, Plexor HY [Auto]/[Y] data may be particularly useful. When a single FFPE sample is available, the calculated [Auto]/[Y] decisional threshold of 5.19, maximizing classification success between highly and mildly degraded STR profiles, can guide laboratory operators in the choice between “miniSTR” and standard STR typing kits. The sensitivity of the decisional threshold of the [Auto]/[Y] measure, i.e., [Auto]/[Y] values <5.19 effectively corresponding to non-degraded samples, was indeed superior in identifying FFPE samples characterized by higher-quality STR profiles, with a specificity of 94.7% compared to the 84.2% shown by [Auto]/[D]. The [Auto]/[Y] decisional threshold can thus effectively orient the selection of the most promising FFPE samples when multiple specimens are available. In paternity cases including multiple FFPE specimens, STR profiles with the highest QS always corresponded to [Auto]/[Y] values <5.19. It must be pointed out that, depending on national guidelines, a minimal number of unambiguously typed STRs (e.g., 15 [49]) and likelihood ratio (paternity index) values above a specific threshold (e.g., 10,000 [50]) may be required for paternity testing. In standard STR kits used to analyze the paternity cases included in this study, the number of loci with an amplicon range <250 bp varied between seven (Investigator ESSplex, PowerPlex ESI 17) and eight (AmpFlSTR Identifiler Plus, PowerPlex ESX 17). Therefore, the use of multiple STR kits, possibly including an additional eight-locus “miniSTR” kit [26] is expected to be mandatory in highly degraded FFPE samples ([Auto]/[Y] > 5.19). Since the combined analysis of ≥15 STR loci is recommended to guarantee paternity indexes >10,000 [50], and partial STR profiles may be observed even in only mildly degraded FFPE samples (QS > 250 bp), the observation of [Auto]/[Y] values <5.19 does not exclude per se the necessity of multiple STR kits to achieve paternity confirmation and simply indicates that the use of specialized miniSTR kits is not justified in this specific case.

The use of [Auto]/[Y] Plexor HY value as a tool to estimate DNA degradation is restricted to male samples. Although this is not a limitation in the context of forensic investigations of deficiency paternity cases, LT-DNA samples of the alleged father may derive from specimens other than FFPE material, such as exhumed cadaver tissues, undergoing patterns of degradation different from those prompted by formalin fixation. It may therefore be of interest in the future to also extend the study of the suitability of the [Auto]/[Y] degradation index to non-FFPE LT-DNA samples. Outside of forensic applications, the present spread of next-generation sequencing technologies makes archival FFPE samples a potential source of endless information in biomedical research, and the precise assessment of FFPE DNA quality is crucial for sequencing applications [51]. Since DNA degradation is a core issue in forensics, qPCR assays originally devised for investigative genetics applications can fit this purpose. However, the actual trend towards the standardization and accreditation of methods in forensics means that last-generation qPCR assays developed by manufacturers to incorporate degradation targets are frequently designed to run on a limited range of RT-PCR platforms [52]. On the other hand, the Plexor HY qPCR system can be implemented on several types of RT-PCR instruments [10,36,48,53,54] and, as demonstrated by the obtained results, can provide a reliable alternative to current non-forensic methods for the prediction of DNA degradation [45] in studies focusing on FFPE specimens of male patients [55] or derived from male-specific tissues [56].

## Figures and Tables

**Table 1:** In cases with *n* > 1 FFPE specimens, QS of STR profiles obtained from the samples with the lowest [Auto]/[Y] and [Auto]/[D] were compared with those of the sample with the best QS (or next best QS, in cases where [Auto]/[Y] or [Auto]/[D] already identified the best QS sample). Gain/loss in terms of QS (Δ) for each qPCR assay and concordance (Y)/non-concordance (N) between qPCR assays in selecting the most promising FFPE sample are shown. Samples with [Auto]/[Y] and [Auto]/[D] values above the predictive threshold for highly degraded DNA (5.19 and 12.58, respectively) are underlined.

Case	*n*	Plexor HY QS (bp)			PowerQuant QS (bp)			Concordance
		[Auto]/[Y]	Best/ Next Best	Δ	[Auto]/[D]	Best/ Next Best	Δ	
1	4	196	184	12	184	196	−12	N
6	2	113	117	−4	117	113	3	N
8	3	319	351	−32	351	319	32	N
9	2	326	150	176	326	150	176	Y
10	2	185	159	26	185	159	26	Y
11	2	165	165	0	165	165	0	N
12	5	227	413	−186	254	413	−159	N
13	2	326	326	0	326	326	0	Y
14	8	195	195	0	195	195	0	N
15	2	195	174	21	174	195	−21	N
16	15	322	322	0	322	322	0	N
17	4	139	99	40	//	//	//	//

## Data Availability

All data supporting the results of this paper can be found in the Supplementary Tables.

## Supplementary Materials

The following supporting information can be downloaded at: https://www.mdpi.com/article/10.3390/genes15050622/s1, Table S1: General characteristics, Plexor HY/PowerQuant quantitation results, and STR profile quality score (QS) values of FFPE samples (*n* = 57) analyzed in the study; Figure S1: A synthetic quantitative measurement of STR profile degradation (QS) was calculated by recording the length in bp of the largest CE peak with an RFU height above the 5% stochastic threshold calculated for the relevant STR amplification kit (in this example, 420 bp for the PowerPlex ESI 17 kit). In the depicted STR profile, in which upper values in allelic labels indicate the estimated size in bp and lower values RFU peak heights, the QS is 191 bp (peak shown by the red arrow); Figure S2: Correlation between year of fixation of FFPE samples (y axis) and, on the x axis: total human DNA concentration (ng/µL) measured in DNA extracts by Plexor HY (A) and PowerQuant (B); Plexor HY [Auto]/[Y] (C) and PowerQuant [Auto]/[D] (D) values; QS values of STR profiles (E); Figure S3: Correlation between total human DNA concentration (ng/µL) (y axis) and (A) [Auto]/[Y] measured by Plexor HY; (B) [Auto]/[D] measured by PowerQuant.
