# Supplementary figures and images for: Assessing DNA Degradation through Differential Amplification Efficiency of Total Human and Human Male DNA in a Forensic qPCR Assay
Source: Genes (Basel). 2024 May 14;15(5):622. doi: 10.3390/genes15050622 (PMC11120943; doi:10.3390/genes15050622)

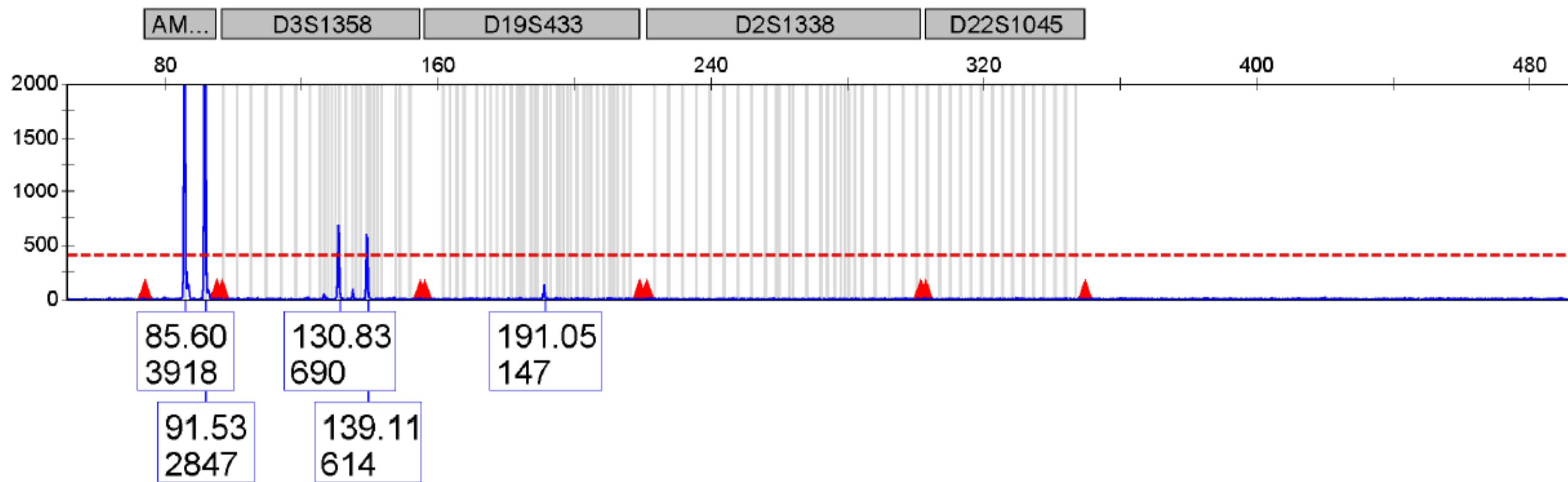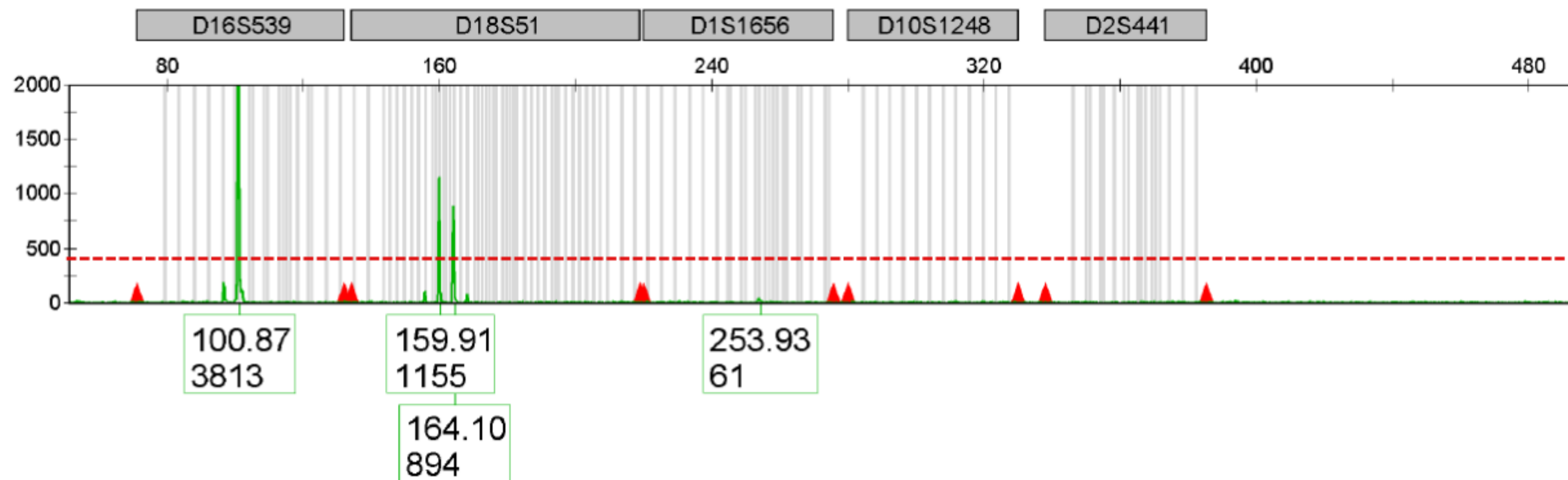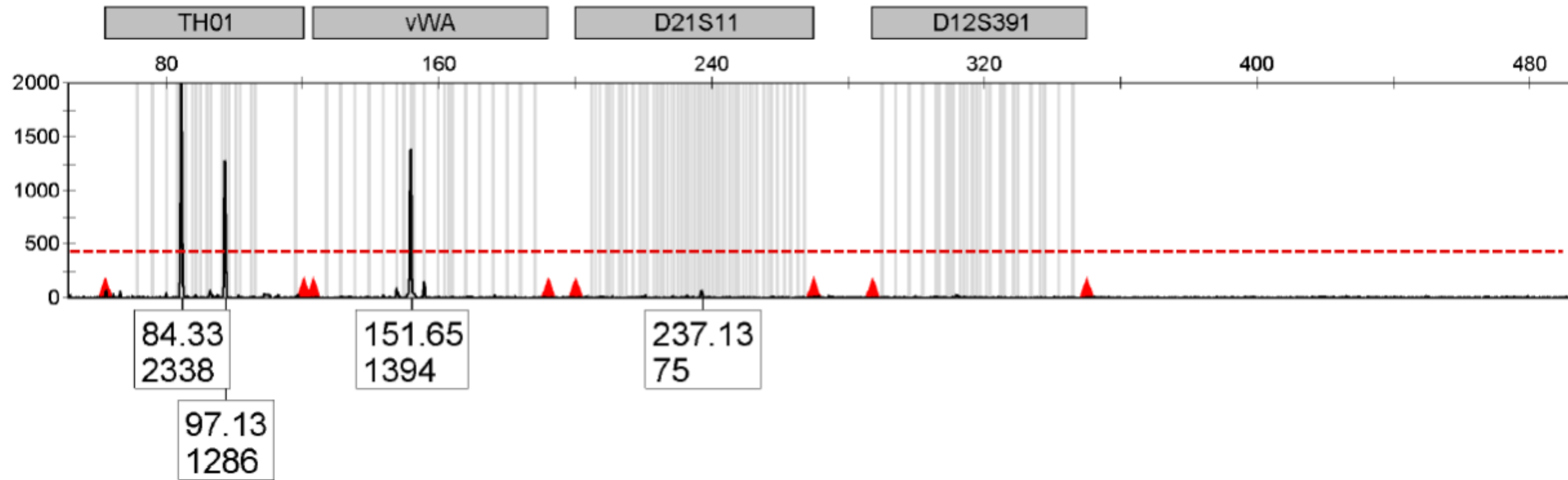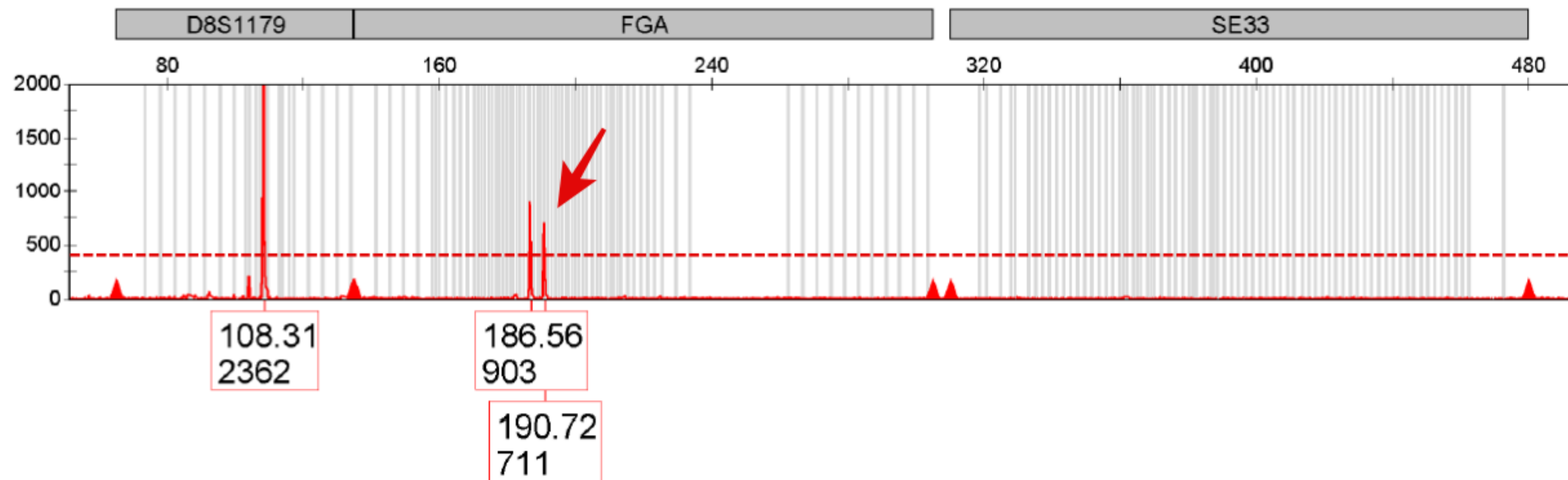

Supplement: Supplementary file 1 [file genes-15-00622-s001.zip › Figure S1.pdf]

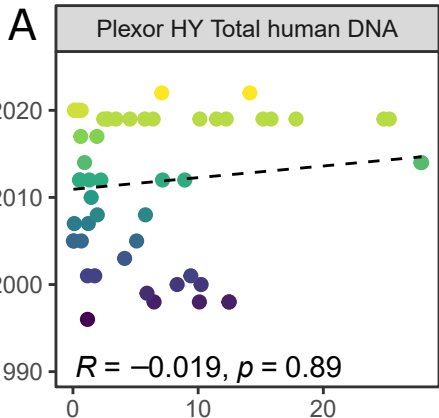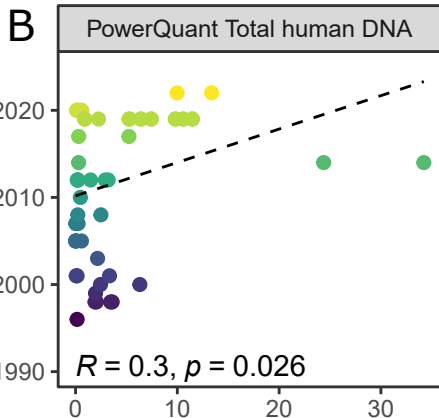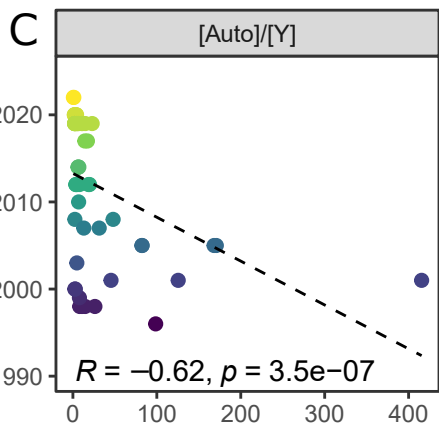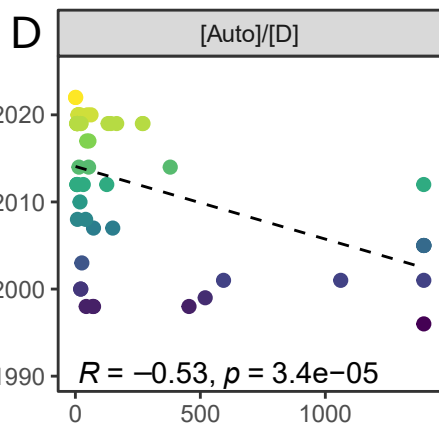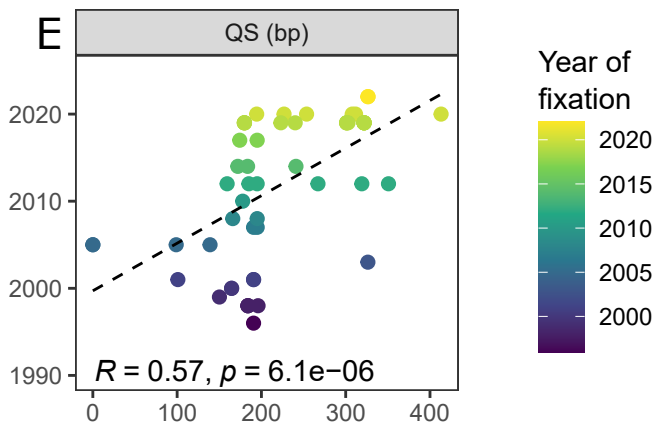

Supplement: Supplementary file 1 [file genes-15-00622-s001.zip › Figure S2.pdf]

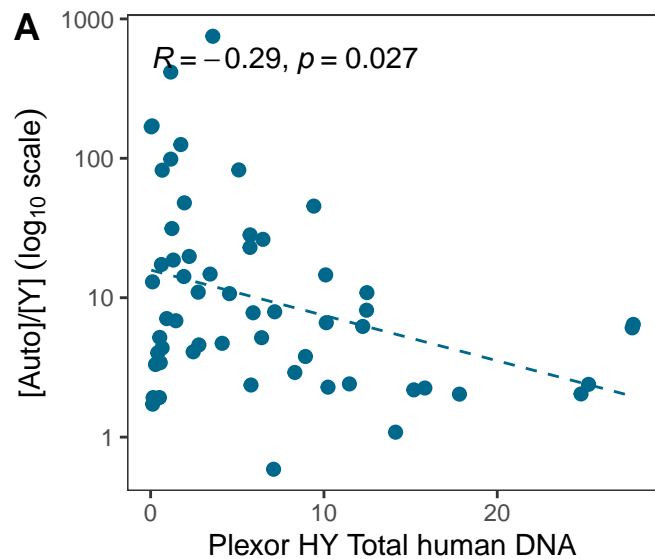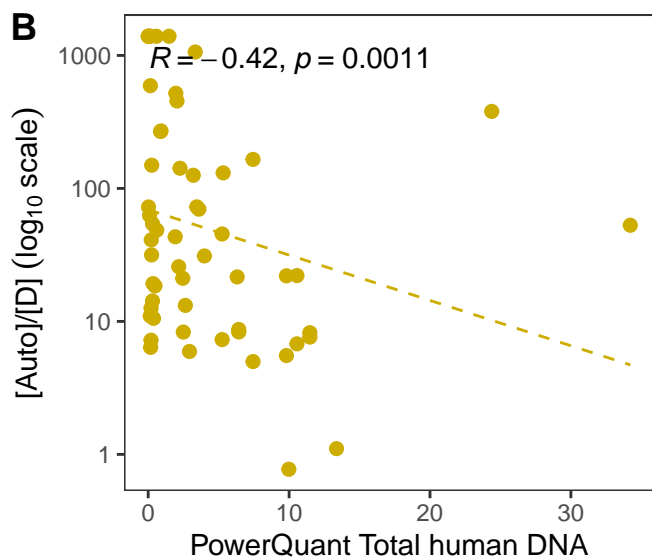

Supplement: Supplementary file 1 [file genes-15-00622-s001.zip › Figure S3.pdf]
